# The data-intensive research paradigm: challenges and responses in clinical professional graduate education

**DOI:** 10.3389/fmed.2025.1461863

**Published:** 2025-02-07

**Authors:** Chunhong Yang, Yijing Chen, Changshun Qian, Fangmin Shi, You Guo

**Affiliations:** ^1^Academic Affairs Office, First Affiliated Hospital of Gannan Medical University, Ganzhou, China; ^2^School of Public Health and Health Management, Gannan Medical University, Ganzhou, China; ^3^School of Information Engineering, Jiangxi University of Science and Technology, Ganzhou, China; ^4^Medical Big Data and Bioinformatics Research Centre, First Affiliated Hospital of Gannan Medical University, Ganzhou, China; ^5^Ganzhou Key Laboratory of Medical Big Data, Ganzhou, China

**Keywords:** data-intensive research, clinical medicine, graduate education, challenges, responses

## Abstract

With the widespread application of big data, artificial intelligence, and machine learning technologies in the medical field, a new paradigm of data-intensive clinical research is emerging as a key force driving medical advancement. This new paradigm presents unprecedented challenges for graduate education in clinical professions, encompassing multidisciplinary integration needs, high-quality faculty shortages, learning method transformations, assessment system updates, and ethical concerns. Future healthcare professionals will need not only to possess traditional medical knowledge and clinical skills, but also to master interdisciplinary skills such as data analysis, programming, and statistics. In response, this paper proposes a series of countermeasures, including curriculum reconstruction, faculty development, developing and sharing resources, updating the evaluation and assessment system, and strengthening ethics education. These initiatives aim to help clinical graduate education better adapt to this new paradigm, ultimately cultivating interdisciplinary talents in medical-computer integration.

## 1 Introduction

The rapid progress of information technology, particularly in big data, machine learning (ML), artificial intelligence (AI), and large language models, is causing a significant transformation in the paradigm of scientific research. This transformation is especially visible in the field of clinical research, ushering in a new, data-intensive research paradigm (1). At the core of this paradigm lies in systematic management and thorough leveraging of real-world data for comprehensive analysis, thus uncovering the complex connections between health and disease. This, in turn, drives the advancement of medical knowledge and the evolution of clinical practice.

In this shifting paradigm, traditional research methods that rely on small-scale sampling and hypothesis-driven approaches are giving way to exploratory research that leverages big data and data-driven methodologies (2). This shift in approach empowers medical researchers to grasp the occurrence, evolution, and therapeutic results of diseases in a more thorough and organized way, providing solid support for precision medicine and tailored treatment.

## 2 Key features of the data-intensive research paradigm

In the field of medical science research, there is an increasing focus on a data-intensive research paradigm, which is revolutionizing traditional methods and processes. This shift is creating new opportunities and directions for the field, highlighting the importance of analyzing and defining its characteristics to predict its future impact.

### 2.1 Deep application of big data

Big data applications have infiltrated numerous facets of the healthcare sector, especially in gathering, analyzing, and clinically applying electronic medical records (3), ergonomic data (4), and information from wearable devices, showcasing considerable real-world value and limitless potential. With the expansion and increasing use of electronic medical record systems, healthcare services have been able to gather, store, and manage the data created by patients during the diagnostic and treatment process more efficiently. The quick gathering of data has supplied an abundance of resources for clinical research, allowing for a more thorough examination of the genetic and molecular underpinnings of human health and diseases. The analysis of multisource, multimodal data presents practical strategies for precision medicine and personalized treatment alternatives.

### 2.2 Rapid progress in artificial intelligence and machine learning

Artificial intelligence and machine learning are revolutionizing the healthcare sector, particularly in disease diagnosis, treatment planning, and supporting clinical decisions (5). By analyzing and learning from extensive real-world patient data, AI and ML can identify early signs of diseases that are difficult to detect. They can also make accurate predictions and early diagnoses when traditional methods fall short. This capability not only significantly improves diagnostic accuracy but also enhances the efficiency of disease diagnosis, enabling patients to receive treatment earlier. When developing treatment plans, AI and ML can swiftly recommend personalized medical advice for patients by analyzing historical patient treatment data and the current patient’s clinical phenotype characteristics (6). This enhances treatment outcomes and minimizes the unnecessary use of medical resources and time. Additionally, the application of AI and ML in clinical decision support aids doctors in making more accurate and efficient decisions in complex clinical situations by offering insights and recommendations based on big data analysis (7). In summary, the integration of AI and ML is gradually transforming the healthcare industry. By improving diagnostic accuracy, optimizing treatment plans, and enhancing the quality of clinical decisions, they provide safer, more effective, and more personalized medical services to patients.

The application of large language models in clinical data management underscores the profound integration of AI technology in healthcare. As an advanced AI technology, large language models can aid medical professionals in achieving more accurate data classification, integration, and analysis by deeply learning and understanding vast amounts of medical text data (8). This not only helps medical institutions effectively manage medical records, research reports, and clinical trial data but also provides robust data and technical support for disease diagnosis, treatment recommendations, and patient care. Additionally, the ability of large language models to process natural language has significantly enhanced the efficiency and accuracy of understanding, analyzing, and structuring unstructured text in medical records. This offers a more solid foundation for further uncovering the value behind the data.

### 2.3 Unprecedented multidisciplinary collaboration

Multidisciplinary integration is an innovative and forward-thinking trend in contemporary research and social practice, especially in the deep integration of fields such as clinical medicine, computer science, and statistics (9, 10). This not only broadens the scope of medical science research but also significantly enhances the efficiency of medical research and the application of its results. The high-quality development of clinical medical practice drives researchers to continuously explore the nature and progression of diseases. Meanwhile, advancements in computing power and algorithms provide robust technical support for processing large-scale health data. Machine learning and statistical methodologies ensure the scientific validity and accuracy of clinical research outcomes. This deep interdisciplinary integration not only advances precision medicine by making disease prevention, diagnosis, and treatment more personalized and accurate but also fosters the widespread application of technologies like artificial intelligence and big data in healthcare, significantly enhancing the quality and efficiency of medical services. Thus, the integration of multiple disciplines has emerged as the primary driving force for advancing medical technology and achieving sustainable, high-quality development in healthcare.

## 3 The transformative impact of data-intensive research paradigm on clinical studies

The influence of data-intensive research paradigm on clinical research is growing more significant by the day. In comparison to conventional methods, data-intensive research paradigm provide more comprehensive and detailed data support, broadening the range, depth, and pace of clinical research. With their distinct advantages, data-intensive research paradigm is revolutionizing clinical research methodology and is emerging as a primary catalyst in propelling medical science forward and improving human health.

### 3.1 Accelerating the evolution of precision medicine

Precision medicine aims to personalize treatment plans for patients by considering their individual characteristics such as genetics, lifestyle, and environment. This approach seeks to improve treatment outcomes and reduce side effects. Transitioning to a precision medicine model involves three key considerations: (1) collecting comprehensive data on patients, including genetic and real-world information, (2) developing advanced analytical tools for patient stratification and prediction, and (3) integrating these tools into clinical practice through medical information systems. This data-intensive research paradigm utilizes big data technologies, high-performance computing, artificial intelligence, and other advanced tools to analyze healthcare data and support precision medicine research effectively.

### 3.2 Prediction and management of risks and benefits in disease treatment

The new paradigm of data-intensive research involves combining multivariate analysis and machine learning to utilize data from various fields like genomics, clinical phenomics, and imaging for creating predictive models. This advancement improves the quality and scope of research on disease treatment risk assessment and interventions, reducing uncertainty in patient prognosis and treatment outcomes. By analyzing individual health data, including genetic information, lifestyle habits, medical history, and environmental factors, machine learning algorithms can develop complex predictive models to accurately predict an individual’s future disease risks (11). These predictions help individuals anticipate potential health risks and treatment responses, while also providing healthcare professionals with scientific evidence to customize more personalized interventions based on the predictive results. This allows for early detection and intervention to improve treatment effectiveness and reduce side effects.

### 3.3 Accelerating new drug discovery and drug repurposing

Data-intensive research is becoming a powerful driving force in the medical field, particularly in the discovery of new drugs (12). By analyzing large amounts of data on drug-target interactions, scientists can quickly and accurately identify new therapeutic targets. This approach also allows for the discovery of potential new uses for existing drugs, which can then be validated through experiments to determine their effectiveness and safety. By utilizing this method, the drug development process can be accelerated, costs can be reduced, and the success rate of research and development can be improved.

Moreover, by extensively exploring current drug databases, not only can new therapeutic uses be uncovered, but robust scientific evidence for drug repurposing can also be obtained (13). This significantly enhances medical resources and expands treatment options for patients. Thus, utilizing data-intensive research in the realm of new drug discovery undoubtedly paves the way for efficient, cost-effective, and innovative research and development, thereby playing a pivotal role in the advancement of pharmaceutical science.

### 3.4 Developing personalized treatment plans

As personalized medicine becomes increasingly important in healthcare, data-intensive research are playing a crucial role in developing individualized treatment plans for patients. This research approach utilizes advanced bioinformatics technologies, big data analysis, and artificial intelligence algorithms to analyze patients’ genetic backgrounds and disease characteristics. By delving into patients’ genetic information, biomarkers, and disease progression, researchers can uncover the underlying mechanisms of diseases and predict how patients will respond to specific treatments. This personalized approach not only allows medical experts to tailor treatment plans to each patient’s unique needs, but also improves treatment outcomes, reduces unnecessary side effects (14), and ultimately achieves true personalized medicine. Additionally, this data-intensive personalized treatment strategy can provide valuable data support for future medical research, driving innovation and advancements in the field of precision medicine.

## 4 Challenges in clinical professional graduate education

The importance of data science and programming skills is on the rise in the healthcare sector. However, traditional medical courses do not focus on developing these abilities. Most medical schools still adhere to traditional curriculum models that emphasize biomedical knowledge and clinical skills, providing limited education on modern medical technologies such as data science and programming.

### 4.1 Interdisciplinary integration of data-associated knowledge and skills

In today’s clinical practice, the integration of knowledge and skills from various disciplines is essential for improving healthcare quality and supporting patient recovery (15). The rapid advancement of data science is blurring the lines between medicine and other fields, leading healthcare teams to adopt a multidisciplinary approach to address complex clinical challenges. Fields such as computer science and data analysis are increasingly becoming indispensable in healthcare, allowing providers to create personalized treatment plans and facilitate knowledge sharing among team members.

The integration of artificial intelligence and big data in healthcare is fostering cross-disciplinary teamwork, providing novel approaches and tools for medical practice, and fueling advancements in healthcare services. Nevertheless, existing educational frameworks are failing to equip medical students with the necessary data processing and analytical capabilities, hindering their capacity to utilize cutting-edge technology in diagnosing illnesses, devising treatment strategies, and enhancing healthcare delivery in their professional journeys.

### 4.2 Urgently needed educational resources and teaching staff

Educators in the medical data science field need a strong foundation in medical practice, as well as expertise in mathematics and statistics. However, there is a lack of high-quality resources for teaching medical data science. There is a shortage of textbooks and course content, as well as a lack of teachers who can effectively teach this material (16). Building a qualified team of medical data science educators is a major challenge, as it requires teachers to have in-depth knowledge of data science, integrate medical expertise, and effectively teach educational concepts and methods.

Furthermore, teachers must continuously update their knowledge base to keep pace with the rapidly evolving field of data science. Thus, forming a diverse and highly qualified team of medical data science educators requires not only systematic training and professional development pathways but also collaborative efforts from educational administrative departments, medical higher education institutions, and the industry.

### 4.3 Outdated teaching techniques

Data-intensive research involves actively uncovering in-depth information within data and exploring unfamiliar subjects. This process goes beyond learning within a single discipline, emphasizing the integration of multiple disciplines. By combining knowledge from various fields, a comprehensive and multi-perspective research approach is developed. This interdisciplinary integration not only fosters innovation and breakthroughs in knowledge but also enhances understanding of complex issues and facilitates finding effective solutions. Therefore, transitioning from passive knowledge acquisition to active exploration and from learning within a single discipline to integrating multiple disciplines is essential for advancing clinical scientific progress and technological innovation in data-intensive research. To succeed in the constantly evolving clinical practice environment and surmount challenges, clinical researchers must possess a diverse knowledge background, a broad perspective, sharp insight, and innovative thinking skills (17). This is because the data-intensive research paradigm requires a focus on developing critical thinking, communication, collaboration, and creativity skills.

### 4.4 The evaluation and assessment system deviates from the genuine requirements

Data-intensive research is becoming more and more important, particularly in real-world studies. Nevertheless, existing assessment system frequently fall short in thoroughly assessing students’ skills in this field. Traditional assessment methods, like standardized tests and course grades in medical statistics, primarily emphasize students’ ability to memorize and comprehend knowledge. However, they do not adequately evaluate students’ skills in identifying which clinical issues can be addressed with existing data and analysis methods or determining the necessary data and methodological support for specific clinical problems. The field of data-intensive research demands students to not only master a vast amount of theoretical knowledge but also possess high levels of logical thinking, critical thinking, and innovation. Evaluating these abilities often requires more in-depth, flexible, and diverse approaches.

As technologies such as big data and artificial intelligence continue to advance rapidly, the demands for knowledge and skills in data-intensive research areas are always changing. This means that assessment system need to be able to adjust to new requirements quickly by updating their content and methods. Therefore, to effectively evaluate students’ abilities in these fields, it is crucial to revamp and modernize the current assessment system, creating more comprehensive, adaptable, and forward-thinking methods that accurately showcase students’ capabilities and potential.

## 5 Response and outlook

In order to meet the needs of clinical professional graduate students training in the era of data-intensive research, it is essential to establish a set of thorough strategies. This includes restructuring the curriculum and enhancing faculty development, sharing and developing resources, and updating evaluation and assessment system. Furthermore, it is crucial to improve the ethical awareness and data protection consciousness of clinical professional graduate students, ensuring they comply with applicable laws, regulations, and ethical standards when managing patient data.

### 5.1 Advancements in medical data science courses and teaching methods

Creating a medical data science course that combines data science, statistics, and programming fundamentals offers students a well-rounded education in data analysis and application. This approach also encourages students to think creatively and identify clinical problem-solving skills. To improve learning outcomes, incorporating case studies and problem-driven learning is essential. Case studies provide real-world examples that help students connect theoretical concepts to clinical practical situations, leading to better retention of memory and increased motivation to learn (18).

**Figure 1:**
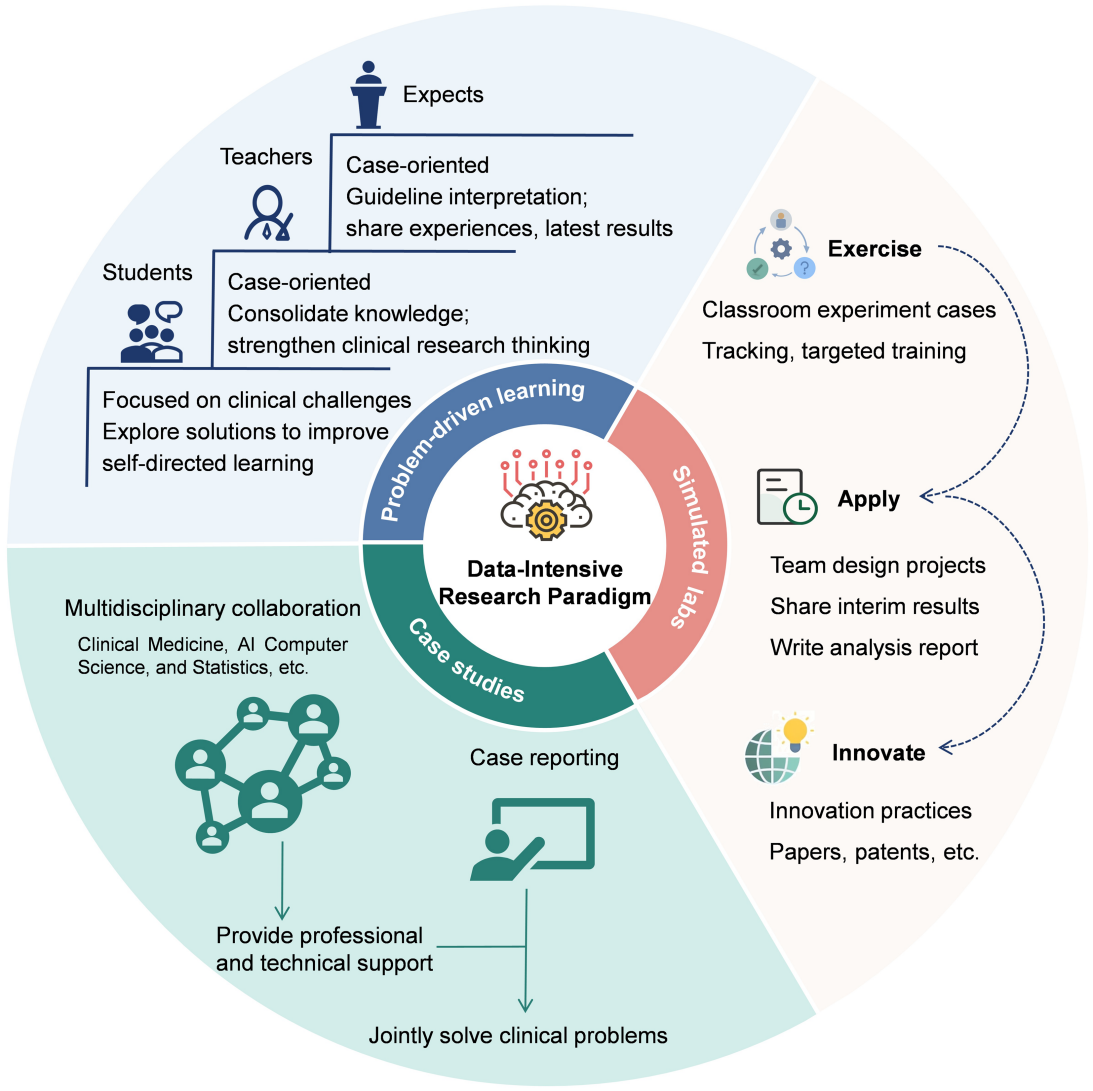
The implementation workflow of problem-driven learning, case studies, and simulated labs in the data-intensive research paradigm.

Problem-driven learning is a teaching approach that emphasizes tackling real-world clinical issues throughout the course (19, 20). This method encourages active participation and helps students develop teamwork, communication, and problem-solving skills. Moreover, the stimulated labs provide students with a comprehensive training platform for pre-course preparation, in-class learning, and post-course consolidation, allowing them to enhance practical skills and foster innovative thinking through the integrated three-dimensional teaching model of “exercise, apply, and innovate” (21). By incorporating various disciplines like data science, statistics, and programming basics, and using case studies, problem-solving activities and stimulated labs (Figure 1), students can improve their learning efficiency and practical abilities. This sets a strong groundwork for their future in the field of data-intensive clinical research.

### 5.2 Building a team of medical data science experts

Establishing and strengthening the teaching staff of Medical Data Science is essential for improving the quality of education and addressing various training requirements. To accomplish this objective, medical colleges and clinical training facilities must adopt a holistic approach. This includes providing thorough and structured training for existing medical data and statistics instructors, as well as actively seeking out new faculty members with varied interdisciplinary backgrounds and extensive real-world experience.

By offering sustainable opportunities for existing medical data and statistics instructors to improve their subject knowledge, they can not only update their thinking and teaching techniques, but also become proficient in the newest educational technology. This can ignite their passion for teaching and research, and boost their appeal, presentation, and impact on student learning.

Bringing in new teachers with varied interdisciplinary educational backgrounds can inject new perspectives and creative teaching approaches into medical schools. This can foster interdisciplinary collaboration and address the growing need for data science skills in clinical settings. Additionally, a diverse teaching team can better reflect the disciplinary diversity of clinical practice, providing students with a broader perspective on clinical medical data.

### 5.3 Sharing and collaborating on learning resources

Unprecedented changes are being witnessed in the field of medical data science education. With the continuous advancement and popularization of Internet technology, online courses, open educational resources, and collaboration among universities have become crucial for driving educational innovation and enhancing quality for clinical professional graduate students. By providing a variety of resources and collaboration models, learners have access to a wide range of materials and practical opportunities. This opens more ways to acquire data knowledge and facilitates personalized and ongoing learning.

The availability of online courses has eliminated barriers related to location, time, and access to crucial clinical data resources, making education and training more convenient and accessible for clinical professional graduate students. By promoting open clinical data resources, these professionals can access high-quality educational materials in clinical data science at little to no cost, including e-books, tutorial videos, and curriculum guides. This not only reduces the financial burden of learning but also facilitates the sharing and dissemination of medical data science knowledge. Additionally, collaborating with non-medical and data engineering subjects provides clinical professional graduate students with opportunities for interdisciplinary and cross-cultural learning. Through resource sharing, joint research projects, and academic exchange activities, this collaboration enhances the learning experience and promotes mutual growth among universities.

Create a customized learning platform that identifies behavioral fingerprints from the study habits of clinical professional graduate students, examining individual learning patterns, interests, and effectiveness. Through the analysis of this information, design personalized learning strategies for each clinical professional graduate students to assist them in mastering knowledge more efficiently and improving their academic skills comprehensively.

To sum up, by utilizing online clinical data science courses, open educational resources, collaborating with non-medical and data engineering disciplines, and creating personalized learning platforms, clinical professional graduate students can benefit from a more accessible, productive, and engaging learning environment. This approach helps in nurturing medical professionals with a strong focus on data-intensive research thinking and skills.

### 5.4 Upgrade the quality assessment system for training clinical professional graduate students

The rapid development in data-intensive research fields has raised higher demands on the overall capabilities of clinical professional graduate students. Therefore, there is an urgent need in the medical education sector to develop new assessment systems and standards that more comprehensively and accurately reflect the abilities of clinical professional graduate students in this field. This involves not only assessing skills such as data analysis, data management, and data interpretation, but also evaluating the innovative ability, critical thinking skills, and teamwork spirit of clinical professional graduate students in addressing real-world clinical challenges. Despite scholars’ innovative efforts, such as integrating capability-driven AI teaching into medical curricula (22) and establishing a multi-platform collaborative “virtual AI laboratory” for dynamic evaluation of classroom experimental projects (which comprehensively cover routine assignments, group projects, and innovation outcomes) (21), resource constraints remain prevalent. Against this backdrop, the AI-driven evaluation platform can be streamlined with open-source frameworks, free cloud computing, and simulation-generated data to cut costs and ease resource strain through local collaboration.

The new assessment system and standards should cover all relevant aspects of capabilities in clinical research while also being flexible enough to adapt to changing needs. Fairness and inclusivity must be key considerations in the assessment process to ensure that all clinical professional students could practice and showcase their abilities. Developing such assessment systems and standards can help guide educational training programs, provide timely feedback to students on their progress, and ensure the equitable distribution of resources in clinical data science education. Collaboration between clinical medical educators, management experts, and data scientists is essential to create a scientifically sound and forward-thinking assessment system and standards through interdisciplinary cooperation, ultimately contributing to the sustainable development of medical education.

## 6 Ethical considerations for data-intensive research paradigm

The rapid development of data science and technology has brought about improvements in efficiency and precision to traditional healthcare. However, it has simultaneously triggered a series of profound and complex ethical issues (23, 24). In essence, these dilemmas are the core wellspring of ethical considerations in the data-intensive research paradigm, meriting our utmost attention. The foremost issue is the challenge of privacy protection. In the process of data acquisition and utilization, although countries are strengthening the regulation of medical data, the pace of technological iteration far outpaces the update of regulations, highlighting a significant contradiction (25, 26). Secondly, the transparency of AI decision-making warrants critical attention. The “black-box” nature of algorithms makes it difficult to clarify the attribution of responsibility in cases of decision-making deviations. The absence of transparency and an accountability mechanism not only endangers patients’ health but also exposes medical institutions and doctors to legal and moral risks. Therefore, it is recommended to construct an interpretable AI framework and establish clear demarcations of responsibility (27).

Furthermore, due to potential data biases, algorithms may generate unfair assessment results (28). This requires educators to transform from traditional knowledge transmitters to learning facilitators and ethical guides. Various teaching strategies, such as case-based teaching (29), embedded AI ethics education frameworks (30), and AI scenario-based teaching in clinical practice (31), can be employed to equip healthcare professionals with both technical skills and moral insights. Meanwhile, the application of AI in clinical education may exacerbate the digital divide. Strengthening ethical regulations and allocating resources rationally are necessary to alleviate this problem (32). Lastly, the issue of doctor-role conflict has also drawn much attention. Defining the boundaries between technology and humans requires joint exploration among medical practitioners, technology developers, and the ethics community. Multi-stakeholder collaboration ensures a healthy, sustainable data-intensive research paradigm for medical progress and human health.

## 7 Discussion

In the current epoch of rapid data science and technology progression, data-intensive clinical research has emerged as a novel research paradigm. Bolstered by a robust technical underpinning, abundant educational resources, and a personalized teaching model, it demonstrates substantial feasibility (33, 34). This paradigm can effectively enhance learning efficiency, optimize teaching outcomes, and improve students’ practical capabilities through personalized learning paths and virtual simulation technologies (35, 36). Nevertheless, this advancement is fraught with challenges. These include data privacy protection, ethical compliance, and the potential erosion of students’ critical thinking abilities. The key focus lies in efficiently managing and analyzing voluminous clinical data, as well as transforming it into valuable insights and guidelines for clinical practice. Achieving this goal requires collaborative efforts from technology developers, governments, educational institutions, educators, and students alike.

To tackle these challenges, it is essential to introduce innovative teaching methods in clinical data science. This includes utilizing problem-driven learning, studying disease case examples, and providing simulated labs to enhance students’ skills in clinical data practice and encourage creative thinking with data. Building a diverse faculty team is crucial, requiring the recruitment of educators with expertise in data science and training existing medical statistics instructors to effectively utilize data science methods and tools. Furthermore, offering high-quality resources for learning clinical data science, such as access to international disease databases and data analysis software, is essential for fostering the education of clinical professional graduate students. Enhancing the assessment system by including criteria related to clinical data governance and analytical abilities can further engage students and encourage innovative thinking. Meanwhile, ethical education should be seamlessly integrated into the curriculum system to strengthen the ethical awareness and data protection consciousness of clinical postgraduate students.

The data-intensive research paradigm undoubtedly places higher competency demands on clinical graduates, necessitating the development of a multidimensional capability structure. To begin with, it is essential to solidify foundational knowledge by thoroughly understanding and applying AI tools, while developing robust data analysis skills to accurately interpret data and evaluate results (37). Furthermore, participation in model optimization and external validation from a critical perspective is necessary. Secondly, proficiency in digital healthcare systems and the integration of Internet of Medical devices is required to enhance service efficiency (38). It is equally important to strictly adhere to medical data security regulations and be equipped to handle information security incidents (39). In addition, cultivating resilience in managing stress is vital for effectively addressing academic and career development pressures, enabling better adaptation to industry changes. Of course, integrating these competencies is by no means an easy task. Implementing AI training in a step-by-step manner may prove beneficial.

Remarkably, given the urgency of patient care, it should focus on specific target groups and promote the implementation of the data-intensive research paradigm in a phased approach. In the initial stage, identify the key groups most likely to benefit from data-intensive clinical research, covering basic disciplines like anatomy, physiology, and clinical specialties such as radiology, ophthalmology, and neurosurgery (40). Leveraging AI-assisted diagnosis and automating administrative tasks can optimize workflows and enhance healthcare professionals’ efficiency. However, the concomitant need for innovative thinking brings new challenges and stress. In response, providing technical training and psychological support (41), and establishing effective feedback and improvement mechanisms are essential steps (42, 43). This promotes a balance between adopting data-intensive clinical research practices and alleviation of stress and burnout among healthcare professionals. By implementing these strategies comprehensively, we can enhance the overall application skills of clinical professional graduate students when faced with data-intensive research challenges. We are confident in our ability to cultivate clinical professional graduate students who not only possess a strong foundation in clinical medical knowledge but also excel in addressing clinical data challenges and making significant contributions to medical innovation research.
